# Cognitive impairment on the Alzheimer's Disease Centers' Uniform Data Set Version 3 Neuropsychological Test Battery in a Peruvian population with progressive supranuclear palsy

**DOI:** 10.1055/s-0046-1824433

**Published:** 2026-06-23

**Authors:** Nilton Custodio, Rosa Montesinos, Belén Custodio, Marco Malaga, Alfonso Uribe, Milagros Nuñez, Pamela Bartolo, Zadith Yauri, Katherine Agüero, Graciet Verastegui, José Huilca

**Affiliations:** 1 Instituto Peruano de Neurociencias, Unidad de Diagnóstico de Deterioro Cognitivo y Prevención de Demencia, Lince, Lima, Perú.; 2 Universidad de San Martín de Porres, Facultad de Medicina, Centro de Investigación del Envejecimiento, Lima, Perú.; 3 Equilibria, Unidad de Investigación, Surco, Lima, Perú.

**Keywords:** Attention, Cognition, Executive Function, Language, Supranuclear Palsy, Progressive, Parkinsonian Disorders

## Abstract

**Background:**

Research on the cognitive profile in progressive supranuclear palsy (PSP) has been scarce in Latin America.

**Objective:**

To outline the demographic, clinical, and cognitive profile of Peruvian PSP patients living in Lima, Peru. We sought to determine the relationship between first clinical symptoms and specific cognitive abilities.

**Methods:**

A cross-sectional study of 34 PSP subjects. We used the Uniform Data Set Version 3 Neuropsychological Battery (UDS3-NB), which possesses normative data for the Peruvian population, to assess global and specific cognitive functions. We also performed correlation analyses to determine the relationship between presenting clinical symptoms (parkinsonism, postural instability, and cognitive impairment) and cognitive functioning.

**Results:**

The mean age was 68 years, and mean schooling was 12.1 years in this cohort. The most common initial clinical symptom was parkinsonism (55.9%), followed by postural instability (23.5%) and dementia (20.6%). Our cohort showed poor performance on global cognition, with selective impairment of processing speed, executive function, and episodic memory. Attentional and visuospatial skills were mildly affected, with partial preservation of naming. There was no significant relationship between the initial clinical symptoms and global cognition, except for a slight correlation between parkinsonism and visuospatial function (r = 0.18; *p* < 0.05).

**Conclusion:**

We found that PSP patients in Peru had extensive impairment in executive function, processing speed, and episodic memory, with relatively preserved naming, consistent with other international cohorts. We did not find a correlation between initial clinical signs and cognitive profile, suggesting that an interdisciplinary approach is needed to evaluate patients with suspicion for PSP. Longitudinal studies are needed to more clearly define the progression of neuropsychiatric symptoms in PSP patients.

## INTRODUCTION

Progressive supranuclear palsy (PSP) is a neurodegenerative disease, characterized by vertical supranuclear gaze palsy, axial rigidity, and postural instability, along with cognitive impairment. 1 The term ‘Richardson's syndrome’ (PSP-RS) now refers to the classically described clinical presentation. 2

The clinical criteria for PSP proposed by the National Institute of Neurological Disorders and Stroke and the Society for PSP are highly predictive of its pathology. However, in a multicenter study of 100 autopsy-confirmed cases of PSP, only 24% had originally presented as PSP-RS, although many others develop this subtype's features over time. 3 The new criteria developed by the Movement Disorder Society (MDS), correctly diagnosed PSP in only 25% at first visit, and 63% at last visit, highlighting this condition is underdiagnosed, possibly due its phenotypic variability. 3 4

Furthermore, patients who have frontal lobar features, such as disinhibition/apathy, may resemble frontotemporal dementia (FTD) and may fall in either PSP or in FTD. This has led to the adoption of the “PSP-FTD complex” for patient's frontal lobe features, occasionally also referred to “frontal-like PSP”. 5 More than half of PSP patients can develop frontal dysfunction, ranging from subjective cognitive impairment to dementia. 6 This can also include difficulty with executive functions (EF). Moreover, patients suffer from reduced verbal fluency, attention, processing speed, and memory. 7

In Latin America, research on the cognitive profile in PSP has been scarce, 8 and too fragmented to explore current practices on FTD. 9 10 Existing studies have primarily focused on specific domains 11 or multidimensional clinical assessment, 8 with limited attention to cognitive impairment in specific populations with PSP. Moreover, Peru has no studies systematically examining how individual cognitive domains are affected in PSP, nor how patterns of initial clinical symptoms correlate with neuropsychological profile. The present study aims to bridge these gaps by characterizing cognitive abilities among Peruvian adults with PSP.

More specifically, our objectives are to report demographic, clinical and magnetic resonance imaging (MRI) characteristics; to describe the cognitive performance based on comprehensive neuropsychological evaluation; and to evaluate the association between initial clinical symptoms and cognitive profile in Peruvian patients with PSP.

We hypothesize that patients with PSP will exhibit significant EF impairments, with an association between initial clinical symptoms and every cognitive domain.

## METHODS

### Participants

A cross-sectional study was conducted in individuals over 50-years-old from the Instituto Peruano de Neurociencias (IPN), between 2019 and 2025. Eligibility criteria included: PSP diagnosis based on clinical criteria, 1 presence of a guardian or caregiver, over 50-years-old, caregiver's ability to complete the questionnaires, spoke Spanish as a primary language, and had a minimum of 4 years of formal education. Caregivers were family members or close friends who had direct contact with the patient for more than 6 hours each day for 3 consecutive months.

The exclusion criteria were individuals with difficulty performing the cognitive tests due to hearing, visual, or other physical problems; speakers of a language other than Spanish. Also, individuals with a diagnosis of mild cognitive impairment (MCI) or dementia due to Alzheimer's disease, vascular dementia, FTD, and dementia with Lewy bodies. 12 Those who were diagnosed/or had symptoms compatible with psychiatric illnesses; concomitant cerebrovascular pathology; and with a history of addiction or substance abuse. Individuals with hypothyroidism, vitamin B12 deficiency, liver disease, chronic kidney disease, neuroinfections, severe cranioencephalic trauma, among others. We further excluded participants who, in the week prior to the evaluation, took any of the following medications: opioids, decongestants, antispasmodic, anticholinergic, antiarrhythmic, antidepressants, antipsychotics, and antiseizures drugs (valproate, phenobarbital, fentanyl, carbamazepine, and levetiracetam). In cases where patients would take these medications, and only if their medical condition would allow it, it was recommended to stop their medication for a week prior to cognitive assessment.

### Measures

#### *Demographic information*

Age, years of schooling, gender, dementia family history, and age of symptom onset was ascertained via self-report. Meanwhile, initial clinical symptoms and time to PSP diagnosis were obtained by neurologists. The initial clinical symptom variable, including parkinsonism, postural instability, and cognitive symptoms, was collected for all patients during the first neurological evaluation.

#### *Brief cognitive and functional screening*

To evaluate cognitive function, we used the Peruvian version of the mini mental state examination (MMSE). 13 This test assesses verbal memory, working memory, language, and visuospatial functions. For functional screening, we used the Pfeffer functional activities questionnaire (PFAQ), composed of 11 items assessing instrumental of daily living (I-ADL). Total scores range from 0 to 33 with higher scores indicating higher functional impairment, 14 whose cutoff points are usable in Peruvian cohorts. 15

#### *Clinical dementia rating (CDR)*

The CDR scale was used for staging of dementia severity: 0 for control; 1 for mild dementia, and 2 for moderate dementia. 16 This scale was administered during an interview with both the participant and their caregiver and was scored independently by two blinded evaluators.

#### *Neuropsychological evaluation*

We used the Uniform Data Set Version 3 Neuropsychological Battery (UDS3-NB) 17 developed by the National Alzheimer's Coordinating Center (NACC), including cognitive domains: attention (digit span forward and backward), processing speed (Trail Making Test Part A [TMT-A]), EF (TMT-B), naming (multilingual naming test [MINT]), phonemic fluency (letters P and M for Spanish-speakers), semantic fluency (animals and vegetables), visuospatial skills (Benson complex figure [BCF]-immediate copy), and learning and memory (craft story 21 [CS-21] immediate and delayed recall, and BCF-delayed recall).

The Spanish adaptation of the UDS3-NB did not reveal significant issues with the initial Spanish translation, except cultural adaption was required for the CS-21, phonemic fluency, and MINT. 18 The IPN patients had been administered UDS3-NB by trained neurologists or neuropsychologists to ensure consistency and reliability in different regional projects, such as Multi-partner consortium to expand dementia research in Latin America (ReDLat). 12 Recently, UDS3-NB has acquired comprehensive normative cognitive data for Peruvian adults across a range of formal education levels. 19

#### *Defining cognitive status*

We define cognitive symptoms as self-experienced or noted by the guardian/caregiver, with persistent decline in cognitive capacity, particularly on executive functions, in comparison with a previously normal cognitive status. The definitions of MCI 20 and dementia 21 were based on the results of MMSE, PFAQ, UDS3-NB, and CDR by consensus.

#### *Classification of participants*

The subtypes were classified based on the MDS's PSP criteria, 1 including PSP-RS, progressive gait freezing (PSP-PGF), parkinsonism (PSP-P), and predominant corticobasal syndrome (PSP-CBS). Due to limited sample sizes, we excluded PSP-CBS participants.

#### *Magnetic resonance imaging (MRI)*

All participants were evaluated with brain MRI before the end of the first week of clinical/cognitive evaluation. Imaging took place at the DPI Center in Lima, using an existing protocol acquired from a 3-Tesla Skyra MR System (Siemens Healthineers AG). Study participants were scanned containing volumetric T1 and fluid-attenuated inversion recovery (FLAIR).

The brainstem was divided into 3 subregions (midbrain, pons, and medulla oblongata) on T1 midsagittal sections by drawing two cutting planes: the first line is between the superior pontine notch and inferior edge of the quadrigeminal plate (line 1), and the second line is parallel to the first through the inferior pontine notch (line 2). The midbrain area (M) was above line 1 (excluding the tectum). The pons area (P) was between the liquor-parenchyma-border of the pons and the anterior liquor-parenchyma-border of the 4 ^th^ ventricle, lines 1 and 2. The M/P area ratio was calculated as the ratio of midbrain area to pons area. 22 Image analysis was performed by two independent raters.

### Statistical analysis

We first summarized descriptive variables using measures of central tendency and dispersion for continuous variables and frequencies for categorical ones. The Shapiro-Wilk test was used to check the normality of continuous variables. Student's independent t-test or Wilcoxon's rank test was used to compare quantitative variables, depending on the normal distribution test results.

Similarly, we obtained the Spearman's correlation coefficients and performed a linear regression between initial clinical symptoms (parkinsonism, postural instability, and cognitive symptom) and every cognitive domain (attention, processing speed, EF, naming, phonemic fluency, semantic fluency, visuospatial skills, and learning and memory) obtained by PSP patients. Subsequently, for significant correlations, linear regression models were performed, adjusting for potential confounders.

### Standard protocol approvals, registrations, and patient consents

The study was approved by the Committee for Medical and Health Research Ethics of the Hospital Nacional Docente Madre-Niño (HONADOMANI) “San Bartolomé” (12360-18). Written informed consent was obtained from all patients and informants enrolled in the study accordance with the Declaration of Helsinki.

## RESULTS

### Demographic, clinical, and MRI characteristics

Our study included 34 individuals, with a mean age of 68 and 53% female (M: 16; F: 18) and a mean of 12.1 (SD: 4.9) years of schooling. Detailed of PSP symptoms onset and time to PSP diagnosis, cognitive, functional and severity of cognitive impairment are presented in Table 1 .

**Table 1:** Demographic, clinical, and MRI characteristics of 34 Peruvians with progressive supranuclear palsy

Characteristics		PSP (n = 34)
Female, n (%)		18 (52.9%)
Age, years, average (SD)		68.4 (7.9)
Schooling, years, average (SD)		12.1 (6.2)
Dementia family history, n (%)		5 (14.7%)
Age of symptoms onset, years, average (SD)		64.9 (7.6)
Time to diagnosis, years, average (SD)		2.8 (2.1)
MMSE, average (SD)		21.4 (4.3)
PFAQ, average (SD)		9.8 (4.8)
CDR, average (SD)		1.7 (0.4)
**MRI**	Hummingbird sign, n (%)	16 (47.1%)
	Midbrain area, mm ^2^ , average (SD)	1.07 (0.4)
	Pons area, mm ^2^ , average (SD)	4.84 (0.5)
	Midbrain/pons area ratio, average (SD)	0.23 (0.4)

Abbreviations: CDR, Clinical Dementia Rating; MMSE, Mini-Mental State Examination; MRI, magnetic resonance imaging; PFAQ, Pfeffer Functional Activities Questionnaire; PSP, progressive supranuclear palsy.

Note: Data were presented in mean and standard deviation, and number (%).

### Performance on cognitive domains based on UDS3-NB

From our total, 85% of patients (n = 29) completed the UDS3-NB, showing low performance in global cognition but particular impairment in speed processing (TMT- A), EF (TMT- B + verbal fluency [FV]) and episodic memory (EM) with mild impairment in attentional and visuospatial tasks, as well as partial preservation of naming. To compare the cognitive performance of PSP participants with that of controls, the normative sample was corrected by age, sex, and schooling in Table 2 . Also, we excluded two patients who completed less than 50% of UDS3-NB, two who did not attend their scheduled appointment, and one who completed the assessment 1 month after undergoing a brain MRI.

**Table 2:** The UDS3-NB neuropsychological measure among 29 Peruvians with progressive supranuclear palsy, comparing with normative sample corrected by age, sex, and schooling

Neuropsychological battery			PSP		Control		
Domain	UDS3-NB	M (SD)	Range	M (SD)	Range
Attention, number span test	Forward, total correct	5.4 (2.3)	2–11	6.5 (2.5)	1–14
	Forward, longest span	5.1 (0.8)		3–8		5.7 (1.4)	3–9
	Backward, total correct	3.7 (1.3)		2–8		5.3 (1.7)	2–11
	Backward, longest span	3.4 (0.8)		1–6		4.1 (1)	2–7
Processing speed, TMT	Part A, time (s)	158.6 (34)	96–180	51.5 (23)	19–150
	Part A, errors	1.2 (1.3)		0–3		0.1 (0.4)	0–3
Executive function, TMT	Part B, time (s)	256.2 (76.2)	98–335	132.8 (66.8)	41–300
	Part B, errors	1.8 (2.1)		0–7		0.8 (1.1)	0–5
Language, naming	MINT, total score	28.4 (5.3)	18–32	29.4 (2.1)	21–32
Language, verbal fluency in 60s	Phonemic, M	9.8 (3.4)	3–20	14.2 (4.4)	2–26
	Phonemic, P	9.3 (3.9)		6–18		12.8 (4.1)	2–28
	Semantic, Animals	10.6 (4.1)		6–21		21.6 (5.2)	8–39
	Semantic, Vegetable	13.9 (4.1)		4–26		17 (4.9)	6–35
Visuospatial	BCF copy, immediate, total score	13.6 (1.3)	9–14	15.9 (1)	10–17
Memory	BCF copy, delayed, total score	10.7 (4.8)	5–22	16.3 (5.9)	3–29
	CS-21, immediate recall, verbatim, total units	10.3 (3.2)		2–14		13.7 (4.6)	1–25
	CS-21, immediate recall, paraphrase, total units	7.8 (3.6)		4–14		12 (2.9)	3–17
	CS-21, delayed recall, verbatim, total units	10.5 (4.3)		0–20		14.4 (5.7)	0–28
	CS-21, delayed recall, paraphrase, total units	8.2 (3.6)		2–20		12.6 (4.8)	0–22

Abbreviations: BCF, Benson complex figure; CS-21, craft story 21; MINT, multilingual naming test; PSP, progressive supranuclear palsy; SD, standard deviation; TMT, trail making test; UDS3-NB, Uniform Data Set Version 3 Neuropsychological Battery.

Note: Higher scores indicate better scores except for the Trail Making Test parts A and B.

### Performance on cognitive domains based on UDS3-NB by clinical subtype PSP

Among the three groups (PSP-RS, -P, and -PGF) the RS one showed the most significant cognitive decrease, while patients with PSP-PGF had the most preserved cognitive function. Furthermore, the PSP-RS group had significantly lower scores on the VF, TMT-A, TMT-B, and EM than PSP-PGF and -P ones. On the other hand, the PSP-P group demonstrated significant reductions in verbal EM, particularly in CS-21, paraphrases of delayed recall ( *p* = 0.05) when compared with PSP-PGF. Regarding naming, no significant differences were found in this study ( Table 3 ).

**Table 3:** Performance of UDS3-NB neuropsychological measure among 29 Peruvians comparison between sub-types of progressive supranuclear palsy

UDS neuropsychological measure	PSP-RS (n = 14)	PSP-P (n = 8)	PSP-PGF (n = 7)	PSP-RS vs PSP-P	PSP-RS vs PSP-PGF	PSP-P vs PSP-PGF
Number Span Test Forward, total correct	4.2 ± 2.1	5.5 ± 1.8	5.6 ± 3.1	0.027*	0.014*	0.178
Number Span Test Forward, longest span	3.9 ± 1.3	4.9 ± 1.5	5.1 ± 2.6	0.024*	0.019*	0.366
Number Span Test Backward, total correct	3.5 ± 2.8	4.2 ± 1.9	4.8 ± 2.4	0.037*	0.029*	0.531
Number Span Test Backward, longest span	3.2 ± 1.6	4.1 ± 2.4	3.9 ± 2.6	0.046*	0.067	0.471
TMT-A, time (s)	149.3 ± 63.5	96.7 ± 47.6	88.1 ± 35.3	0.003**	0.001**	0.753
TMT-A, errors	3.4 ± 0.9	3.2 ± 1.3	3.6 ± 0.7	0.408	0.503	0.681
TMT-B, time (s)	201.7 ± 89.3	198.6 ± 93.7	188.8 ± 103.5	0.873	0.602	0.762
TMT-B, errors	5.2 ± 1.8	4.9 ± 2.1	5.3 ± 1.7	0.517	0.926	0.612
MINT, total score	25.3 ± 2.4	26.1 ± 3.1	24.6 ± 3.3	0.812	0.745	0.802
Phonemic Fluency, M, total in 60s	5.1 ± 2.1	6.4 ± 1.9	7.9 ± 1.8	0.006**	0.001**	0.506
Phonemic Fluency, P, total in 60s	4.9 ± 3.5	6.1 ± 2.2	6.7 ± 2.8	0.003**	0.004**	0.405
Semantic Fluency, Animals, total in 60s	7.1 ± 1.7	11.6 ± 3.1	11.8 ± 1.1	0.003**	0.008**	0.234
Semantic Fluency, Vegetable, total in 60s	6.9 ± 2.1	8.5 ± 2.4	9.9 ± 1.8	0.008**	0.002**	0.251
BCF Copy, Immediate, total score	10.1 ± 3.7	11.6 ± 1.5	12.1 ± 2.9	0.013*	0.025*	0.571
BCF Copy, Delayed, total score	9.1 ± 4.4	12.6 ± 1.7	13.2 ± 3.5	0.003**	0.001**	0.481
CS-21, Immediate Recall, Verbatim, total units	7.8 ± 3.6	11.7 ± 1.5	11.1 ± 2.1	0.004**	0.007**	0.361
CS-21, Immediate Recall, Paraphrase, total units	6.7 ± 2.8	12.4 ± 2.1	11.3 ± 2.5	0.001**	0.004**	0.453
CS-21, Delayed Recall, Verbatim, total units	8.1 ± 2.7	12.5 ± 3.1	12.4 ± 3.8	0.005**	0.004**	0.671
CS-21, Delayed Recall, Paraphrase, total units	6.2 ± 1.9	11.3 ± 1.5	13.1 ± 2.6	0.002**	0.001**	0.054*

Abbreviations: BCF, Benson complex figure; MINT, multilingual naming test; PGF, progressive gait freezing; PSP, progressive supranuclear palsy; PSP-P, progressive supranuclear palsy parkinsonism; RS, Richardson's syndrome; TMT, trail making test; UDS3-NB, Uniform Data Set Version 3 Neuropsychological Battery.

Notes: * *p* < 0.05; ** *p* < 0.01.

### Association between initial clinical symptoms and cognitive domain

The initial clinical symptoms from 34 Peruvians with PSP were parkinsonism in 55.9% (n = 19); postural instability in 23.5% (n = 8); and cognitive symptom in 20.6% (n = 7). Overall, we found no association between initial clinical symptoms and every cognitive domain measured by UDS3-NB; except for the PSP-P group, which showed a very weak positive correlation with visuospatial skills (r = 0.18; *p* < 0.05).

## DISCUSSION

In this study, PSP Peruvian patients showed low performance in global cognition but particular impairment in speed processing, EF and EM, but contrary our hypothesis we found no association between initial clinical symptoms and every cognitive domain.

### Demographic, clinical, and MRI characteristics

The sample studied has a median education level, which is consistent with studies conducted in Lima on dementia 22 and FTD. 23 24 This also coincides with previous studies on PSP conducted in Latin America, 11 as usually occurs in middle age, with onset around the age of 65. 24

The mean age (68.4 ± 7.9 years) and time to diagnosis (2.8 ± 2.1 years) of PSP in our study were similar to the German 7 and Brazilian studies, 11 but lower than participants of the Alzheimer's Disease Neuroimaging Initiative (ADNI, 5.3 ± 3.9 years). 25

The global cognitive performance and dementia severity measured by MMSE and CDR were similar to the ADNI, 25 German, 7 Korean, 6 and Chinese 22 studies, but lower than the Brazilian study. 11

The mean M/P area ratio in MRI showed atrophy (0.23) similar to the Chinese 22 and Italian 26 cohorts. Meanwhile, the hummingbird sign (HBS) was found in in 47% of Peruvian patients with PSP. 27 Measuring sagittal midbrain area is more accurate and reliable than visual assessment (HBS, morning-glory, or Mickey-Mouse signs); 27 however, a recent Brazilian research measuring both quantitative and qualitative rates of midbrain atrophy provided modest accuracy in distinguishing PSP from behavioral variant FTD distinguishing by HBS and MP area ratio, with a 73 and 75% accuracy, respectively. 28 Due to significant overlap in appearance, a midbrain with a HBS or reduced sagittal area should raise the suspicion of PSP only after other signs of idiopathic normal pressure hydrocephalus have been considered. 22 27 The mean M/P area ratio was smaller in PSP than in Parkinson's disease, multiple system atrophy, or controls; the change in the midbrain volume was correlated with disease progression, measured by the PSP rating scale. 22 29

### Performance on cognitive domains based on UDS3-NB

In accordance with previous studies, 4 6 22 30 a significant cognitive decline was observed in this PSP cohort, particularly in EF and EM. The EFs measured by TMT- A, TMT-B, and VF were the most impaired cognitive domains. They were associated with pronounced neurodegeneration in the right frontal and temporopolar white matter. 7 From the perspective of secondary care in general neurology, Montreal Cognitive Assessment (MoCA) and Frontal Assessment Battery (FAB) are good options, as they assess EF, disinhibition and aphasia. 31

Interestingly, utilizing subscores (motor series and VF) rather than the total FAB score might be more beneficial for detecting cognitive decline in PSP patients. 32 Recently the Short Cognitive and Neuropsychiatric (ShoCo) scale showed good correlation with MoCA and significant annualized change within the PSP patients (baseline: 6.2 ± 2.9; follow-up: 6.9 ± 3.1; annualized difference: 1.0 ± 3.1; n = 57; *p* = 0.022), concluding that the ShoCo scale seems a promising and valid tool to measure specific neuropsychological disabilities of PSP patients in clinical routine and research. 33

In our cohort, both PSP-RS and -P showed significantly lower word counts than PSP-PGF. Functional MRI studies have demonstrated that both the frontal and temporal lobes play a crucial role in semantic fluency. Furthermore, PSP patients have been reported with greater impairment in phonemic than semantic fluency. As with prior studies, this research confirmed that phonemic fluency is more impaired in PSP-RS than in -PGF. 31 While there was no statistically significant difference in VF between PSP-P and -PGF, -RS tended to have lower word counts, highlighting a greater impairment. The phonemic and semantic fluency assessments remain practical tools for differentiating PSP subtypes.

In the last years, memory decline has repeatedly been described in PSP. 7 11 30 33 The initial observations suggest that it depends on EF and can be caused by deficits in encoding and retrieval operations. 11 More recently, however, new evidence suggests that EM impairment in PSP is also linked to storage deficits due to hippocampal atrophy. 7 11 A strong correlation between memory impairment with abnormalities in temporo-mesial regions and the right medial thalamus has been reported; these are associated with cortical limbic neurodegeneration, especially in the hippocampus. 7 31

Interestingly, attentional tasks (number span test forward and backward), particularly in PSP-RS and -P, were slightly affected in our cohort, reflecting early dementia's stage. This is comparable to the Brazilian cohort that showed preserved attention in participants with MMSE in very early stages. 11 In longitudinal studies, attentional tasks declined after 6 months on follow-up measured by the repeatable battery for the assessment of neuropsychological status (RBANS), 34 number span test forward and backward, 2 or MoCA's subdomain. 7 30

There is evidence that attention is associated with widespread subcortical neurodegeneration with a frontotemporal emphasis. 7 Similarly, the visuospatial task measured by total score of the BCF's immediate copy were mildly impaired in the PSP-RS and -P groups, according with early dementia stages on our cohort. However, an ideal performance on the visuospatial domain requires ocular-motor and motor function for the drawing tasks. As such, there have been attempts to address this by developing a cognitive composite battery for PSP that accounts for the confounding effects of motor impairment. 30

The partial preservation of naming based on MINT is similar to previous reports. A mild naming impairment has been reported when an extended naming task is used, such as the full 60-item version of the Boston naming test; while some studies using shortened naming tasks have found performance to be normal or only mildly impaired. This may suggest that the problem relates more to sustained attention than to the processes required for object naming per se. 35

This study has important strengths. First, we analyzed a well-characterized sample of PSP Peruvian evaluated by a validated neuropsychological tool (UDS3-NB). We also avoided using only brief cognitive screening tools or the RBANS, as they don't extensively evaluate EF. 34 The MoCA can be used to detect cognitive deficits in PSP baseline patients and is superior to the MMSE, but the evidence showed a lack of the sensitivity to changes within the MoCA score and lack of specificity for PSP patients. 30 Second, all diagnoses were established through comprehensive neuropsychological evaluations following standardized criteria, 36 which strengthens their validity.

We acknowledge several limitations and outline directions for future research. The cross-sectional design of our study captures a single snapshot in time. Longitudinal studies are needed to confirm the trajectory of cognitive domain losses in PSP. 31 Given that PSP is generally known to progress more rapidly than other neurodegenerative diseases, 37 a prospective follow-up would likely reveal even more pronounced divergences in how quickly cognitive abilities deteriorate.

Following patients over time could also establish whether specific early cognitive deficits predict subsequent decline or the conversion from mild cognitive impairment to dementia. On the other hand, we didn't include apathy or disinhibition questionnaires, which can be particularly useful in differentiating it from other frontotemporal disease.

In this sense, we didn't include assessments of social cognition, a frontal function previously impaired in Brazilians patients with PSP and FTD; 38 and no biological biomarkers, such as cerebrospinal fluid biomarkers or amyloid positron emission tomography (PET), were evaluated in our study, limiting the confirmation of underlying neuropathologies, and discussion about potential influence of Alzheimer copathology on the cognitive performance of PSP patients. 25

Finally, the use of PFAQ to evaluate functional abilities via caregiver couldn't discriminate impairments of ADL due to motor symptoms or cognitive decline. It is possible that the PSP-rating scale could reflect the cognitive changes in relation to duration and disease severity, including the I-ADL changes. 39

In conclusion, the present study found that PSP patients in Peru had extensive impairment in EF, processing speed, and EM with relatively preserved naming, consistent with other international cohorts. We did not find a correlation between initial clinical signs and cognitive profile, suggesting that an interdisciplinary approach is needed to evaluate patients with suspicion of PSP.
